# Evaluation of Deformable Image Registration Methods for Dose Monitoring in Head and Neck Radiotherapy

**DOI:** 10.1155/2015/726268

**Published:** 2015-02-11

**Authors:** Bastien Rigaud, Antoine Simon, Joël Castelli, Maxime Gobeli, Juan-David Ospina Arango, Guillaume Cazoulat, Olivier Henry, Pascal Haigron, Renaud De Crevoisier

**Affiliations:** ^1^Université de Rennes 1, LTSI, Campus de Beaulieu, 35000 Rennes, France; ^2^INSERM, U1099, Campus de Beaulieu, 35000 Rennes, France; ^3^Centre Eugene Marquis, Radiotherapy Department, 35000 Rennes, France

## Abstract

In the context of head and neck cancer (HNC) adaptive radiation therapy (ART), the two purposes of the study were to compare the performance of multiple deformable image registration (DIR) methods and to quantify their impact for dose accumulation, in healthy structures. Fifteen HNC patients had a planning computed tomography (CT0) and weekly CTs during the 7 weeks of intensity-modulated radiation therapy (IMRT). Ten DIR approaches using different registration methods (demons or B-spline free form deformation (FFD)), preprocessing, and similarity metrics were tested. Two observers identified 14 landmarks (LM) on each CT-scan to compute LM registration error. The cumulated doses estimated by each method were compared. The two most effective DIR methods were the demons and the FFD, with both the mutual information (MI) metric and the filtered CTs. The corresponding LM registration accuracy (precision) was 2.44 mm (1.30 mm) and 2.54 mm (1.33 mm), respectively. The corresponding LM estimated cumulated dose accuracy (dose precision) was 0.85 Gy (0.93 Gy) and 0.88 Gy (0.95 Gy), respectively. The mean uncertainty (difference between maximal and minimal dose considering all the 10 methods) to estimate the cumulated mean dose to the parotid gland (PG) was 4.03 Gy (SD = 2.27 Gy, range: 1.06–8.91 Gy).

## 1. Introduction

Large anatomical variations can be observed during the seven weeks of head and neck cancer (HNC) intensity-modulated radiation therapy (IMRT) treatment course, such as weight loss [1, 2], primary tumor shrinking [1], parotid gland (PG) volume reduction [3], and reduction of the neck diameter [4, 5]. Consequently, the actual delivered dose does not correspond to the optimized pretreatment planned dose, with a risk of overdose of the organ at risk (OAR), in particular the PG [6, 7]. Indeed, if IMRT has been shown, compared to non-IMRT techniques, to dramatically decrease the dose in the PG and therefore the risk of xerostomia [8–10], the benefits of IMRT are however likely reduced by these anatomical variations [6, 7]. Setup errors are relatively easily corrected by a simple rigid registration based on the bony structures. Volume and shape variations can be compensated by replanning, therefore implying to perform new computed tomography (CT) scans during the course of treatment, since the CBCT cannot be yet straightly used for replanning. This recent adaptive radiotherapy (ART) strategy appears however complex, in particular to decide when and how many times to replan during the treatment course [11, 12]. In this context, a key step is the capability to monitor the cumulated dose received by the deformed OARs, fraction after fraction, then to compare this dose with the planned dose, and finally to decide whether or not to replan within a dose-guided adaptive radiotherapy strategy [13, 14].

**Figure 1:**
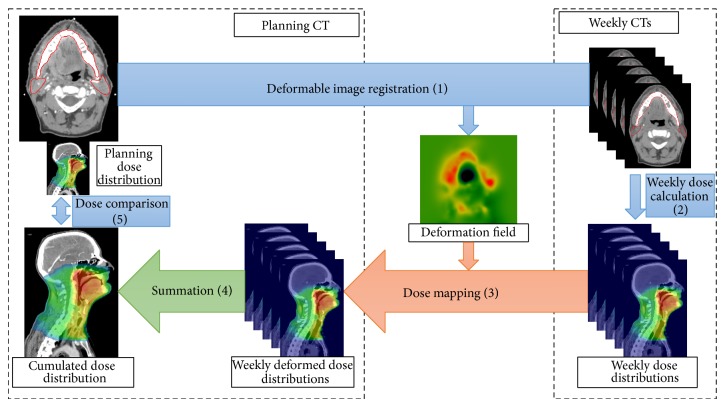
Full cumulated dose scheme in 5 steps. CT: computed tomography (scan).

Deformable image registration (DIR) is a keystone of the dose accumulation process (Figure 1). Different DIR methods have been proposed to register HNC X-Ray CT images [15, 16]. Particularly, the two most popular DIR methods, demons' and the free form deformation (FFD) methods have been considered. Demons' method computes a dense deformation field, which is smoothed to provide a regular field [17]. The FFD approach deforms a grid of a limited number of control points, whose displacements are interpolated using generally a B-spline function to provide a dense field [18]. Thus, these motion regularization approaches are very different. Moreover, other important choices have to be made when using DIR. The similarity metric, used to quantify the matching quality between voxels, is a decisive option and is highly dependent on the considered images. The mean squared error (MSE) is the most classical metric when dealing with monomodal images, and the mutual information (MI) when registering images with different intensity ranges. In the case of HNC CTs, if the MSE seems to be a natural choice, the MI could be useful when the injection of a contrast agent modifies some tissues' intensities in only a subset of the images set. Some preprocessing steps may also be applied to the images before their registration. Indeed, the most challenging task of DIR in the considered application is to estimate the deformation of soft tissues showing a poor contrast. Using some filters to enhance these tissues or even using delineations to guide the registration may be helpful to improve the results.

If the use of DIR appears useful for contour propagation [19, 20], its use to cumulate the dose is a lot more controverted [21–24] and is therefore not yet validated in a clinical practice. Indeed, one of the key issues is the evaluation of the performance of the DIR methods. Volume-based criterion (i.e., dice similarity coefficient (DSC)) can be used [15], providing however no information regarding the registration's anatomical “point to point” correspondence precision. Landmarks (LMs) manually placed by two observers allow to quantify the local accuracy and precision of DIR, while including the intraobserver error [25].

In the context of IMRT for locally advanced HNC, based on weekly CTs in a series of 15 patients, the objectives of this study were to quantify

- the geometrical local accuracy and precision of different DIR methods, including images with or without preprocessing and different similarity metrics;
- the dosimetric impact of using these different methods for dose accumulation in the OARs, in particular the PG.

## 2. Materials and Methods

### 2.1. Patients and Tumors

The study enrolled a total of fifteen patients with a mean age of 65 years (ranging from 50 to 87 years). All tumors were locally advanced head and neck carcinoma (Stage III or IV, AJCC 7th ed), thirteen of them were located in the oropharynx, one in the larynx and one in the hypopharynx. The mean PG volume was 25.3 cc (ranging from 16.6 cc to 52.1 cc, standard deviation (SD) = 8.1 cc).

### 2.2. Treatment and Planning

All patients underwent IMRT delivering a total dose of 70 Gy (2 Gy/fraction/day, 35 fractions), with a simultaneous integrated boost technique [26] and concomitant chemotherapy. Planning CTs (CT0) with intravenous contrast agents were acquired with 2 mm slice thickness, from the vertex to the carina. A thermoplastic head and shoulder mask with five fixation points was used. PET-CT and MRI coregistration was used for tumor delineation. Three target volumes were generated. Gross tumor volume (GTV) corresponded to the primary tumor along with involved lymph nodes. Clinical target volume 70 Gy (CTV_70_) was equal to GTV plus a 5 mm 3D margin, which was adjusted to exclude air cavities and bone mass without evidence of tumor invasion. CTV_63_ corresponded to the area at high-risk of microscopic spread, while CTV_56_ corresponded to the prophylactic irradiation area. GTV, CTV_63_, CTV_56,_ and all organs at risk were manually delineated on each CT slice. Adding a 5 mm 3D margin around the CTVs generated the PTVs. PTV expansion was limited to 3 mm from the skin surface in order to avoid part of the build-up region and to limit skin toxicity [27]. All IMRT plans were generated using Pinnacle V9.2. Seven coplanar 6-MV photon beams were employed with a step and shoot IMRT technique. The prescribed dose was 70 Gy to PTV_70_, 63 Gy to PTV_63,_ and 56 Gy to PTV_56_. The collapsed cone convolution/superposition algorithm was used for dose calculation. The maximum dose within the PTV was 110% (D2%). The minimum PTV volume covered by the 95% isodose line was 95%. Dose constraints were set according to the GORTEC recommendations [28]: a mean dose (*D*_mean_) < 30 Gy and a median dose < 26 Gy for contralateral PGs.

Patients were treated as planned on CT0 and no changes were applied to dose distribution during treatment. During the treatment course, weekly in-room stereoscopic imaging corrected set-up errors > 5 mm. All patients signed an informed consent form.

### 2.3. Per-Treatment Imaging and Volume Variations

**Figure 2:**
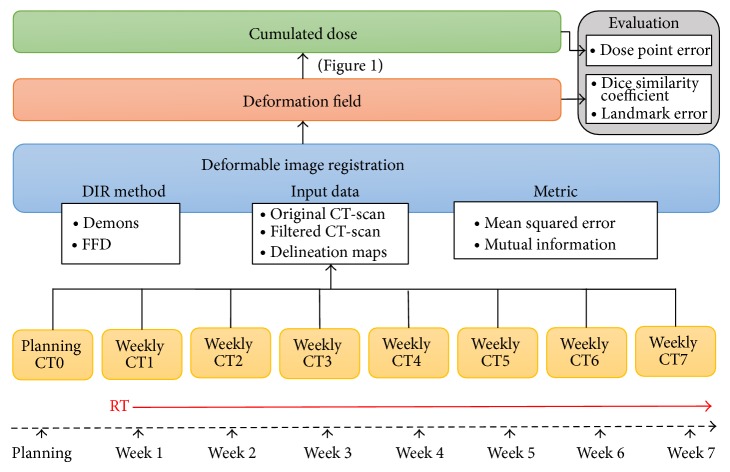
Treatment and workflow study scheme. DIR: deformable image registration, FFD: free form deformation, CT: computed tomography (scan), and RT: radiotherapy.

During the treatment, each patient underwent six weekly CTs (CT1 to CT6) (Figure 2) according to the same modalities as CT0, except for the intravenous contrast agents (not systematically used, particularly in case of cisplatin based chemotherapy). For each patient, the anatomical structures were manually segmented on each weekly CT by the same radiation oncologist. In case of complete response, initial macroscopically involved areas were still included in the CTV_70_, which was adjusted to exclude any air cavities and bone mass without evidence of initial tumor invasion.

**Figure 3:**
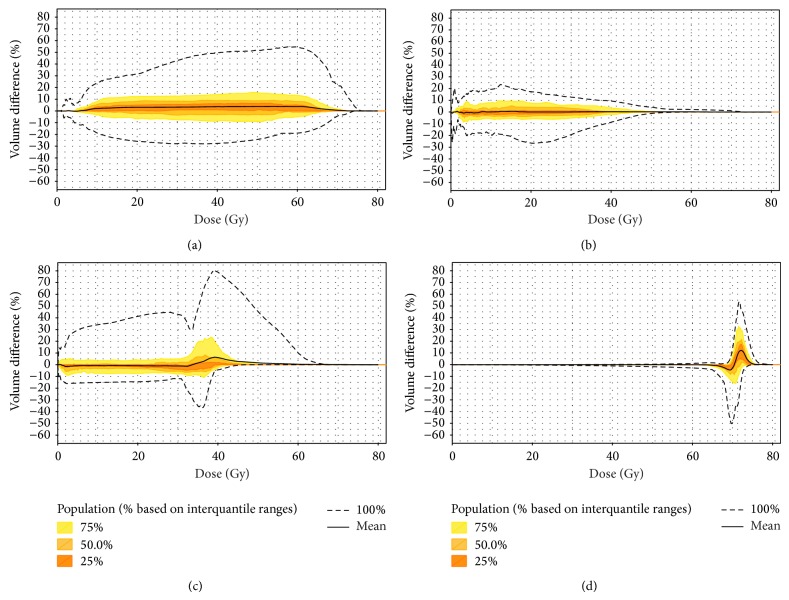
Representation of the DVH differences between the DVH calculated from the weekly CT and the DVH from the planning CT for (a) the parotid glands, (b) the brain stem, (c) the spinal cord, and (d) the CTV_70_. DVH: dose-volume histogram and CTV_70_: clinical target volume receiving 70 Gy. For each subfigure the dashed lines represent the maximum and the minimum DVH difference values; the black line represents the mean DVH difference. Each color represents the 75%, 50%, and 25% interquantile range of the data.

A total of 102 CT scan have been used in the study, 15 planning CTs and 87 weekly CTs.  Figure 3 represents the differences between the DVH from each weekly CT scan and from the planning CT (CT0), for the parotid glands, the spinal cord, the brain stem, and the CTV_70_. The dose at each fraction is normalized to the total dose (70 Gy). The 75%, 50%, and 25% interquantile ranges are represented by colored ribbons.

From CT0 to CT6, the PG volumes decreased by a mean value of 28.3% (ranging from 0.0 to 63.4%, SD = 18%), corresponding to an average decrease of 1.1 cc/week (ranging from 0.0 to 2.2 cc/week). The CTV_70_ decreased by a mean value of 31% (ranging from 73% to −13%, SD = 28%). The thickness of the neck decreased for 78% of the patients by a mean value of 7.9 mm (ranging from 0.1 to 26.6 mm, SD = 6.2 mm).

### 2.4. Cumulated Dose Estimation

Dose accumulation is defined by the addition of the dose distributions at different fractions (in this case from weekly CTs) reported to the planning CT, by applying to the dose distribution at the fraction, thanks to elastic registration, the anatomical transformation from the fraction anatomy to the planning anatomy. The dose accumulation process relied on four steps, as illustrated in Figure 1. Firstly, for each weekly CT, the anatomical variations with respect to the planning CT were estimated using DIR (step 1). The actual weekly dose distribution (step 2) was estimated by calculating the dose distribution on the weekly CT, using the treatment parameters and the CT0 isocenter. The deformation field resulting from DIR was then applied to the corresponding weekly dose distribution to report it to the planning CT (step 3). Finally, all the propagated dose distributions corresponding to all weekly CTs were summed to compute the cumulated dose (step 4). To select the appropriate DIR method, some choices had to be made, especially (Figure 2): (i) the registered images (i.e., with or without preprocessing); (ii) the DIR approach; (iii) the considered metric. In this study, a total of 10 combinations of these criteria were evaluated.

#### 2.4.1. Registered Images

To register each weekly CT to the planning CT, both original images (without any preprocessing) and images resulting from two preprocessing methods (sigmoid filtering and delineation mapping) were considered.

Both planning CT and weekly CT images were preprocessed with a sigmoid filter to enhance soft tissues of the images. Sigmoid filter allowed increasing intensity values between an arbitrary intensity range and decreasing outside that defined intensity range:

(1) $$\begin{array}{c} I^{\prime }=\left(\mathrm{Max}-\mathrm{Min}\right)*\frac{1}{1+e^{\left((I-\beta )/\alpha \right)}}+\mathrm{Min}, \end{array}$$

where *I*′ represent the intensity *I* transformed in the intensity value window [Min; Max]. The sigmoid parameters *α* and *β* define, respectively, the width of the input intensity range and the intensity around which the range is centered. The value of *α* was 100 Hounsfield units (HU) and *β* was 0 HU, corresponding to the intensity range of soft tissues. The Min and Max intensity values were, respectively, −1024 HU and 2976 HU.

In order to focus the registration process on the considered organs, delineation maps were also generated, representing the main delineations (skin, left, and right parotid glands, spinal cord, and brain stem) with homogeneous intensities. The assigned intensity values were (1) for the skin, (2) for the right parotid, (3) for the left parotid, (4) for the brain stem, and (5) for the spinal cord.

#### 2.4.2. Deformable Image Registration Methods

Two DIR methods have been evaluated: demons' method and the free form deformations (FFD).

Demons' algorithm [17] is a nonparametric method which iteratively estimates a dense deformation field (i.e., defined on each voxel) between two images. At each iteration, the deformation field is updated by computing local forces using the intensity gradient and the difference of the intensities of the two images. Then, the deformation field is smoothed using a Gaussian filter (*σ* = 1 or *σ* = 2.5). A multiresolution process, including three levels, was used.

The FFD registration method is a parametric registration method defining a deformation field by an underlying mesh of control points. The control points are iteratively displaced according to the considered metric. The dense deformation field is obtained by a B-spline interpolation [18]. A multiresolution process, including three levels, was used. At each iteration, the adaptive stochastic gradient descent (ASGD) [29] was used to optimize the control point displacements defined by

(2) $$\begin{array}{c} x_{k+1}=x_{k}-\alpha_{k}\nabla f\left(x_{k}\right), \end{array}$$

where *α*_*k*_ represents the step size on *f* along the negative gradient direction.

The good definition of the gain *α*_*k*_ for the convergence of the ASGD is defined by

(3) $$\begin{array}{c} a_{k}=\frac{a}{{\left(K+A\right)}^{\alpha }}, \end{array}$$

with *a* = 10^4^, *A* = 51.0, and *α* = 0.602 for head and neck registration. The resolution of the grid was chosen at 30 mm, 20 mm, and 10 mm and then 5 mm for the last resolution.

#### 2.4.3. Metrics for DIR

Two metrics were considered in the DIR process: the mean squared error (MSE) and the mutual information (MI).

The mean squared error is defined by

(4) $$\begin{array}{c} \text{MSE}\left(A,B\right)=\frac{1}{N}*\overset{N}{\underset{i=1}{\sum }}{\left(A_{i}-B_{i}\right)}^{2}, \end{array}$$

where *A*_*i*_ and *B*_*i*_ are the intensity of the *i*th pixel of the *A* and *B* images and *N* is the number of considered pixels. This metric was used in conjunction with the three types of images (original images, original images filtered by the sigmoid filter, and delineation maps) and the two registration methods (demons and FFD).

The mutual information is defined by

(5) $$\begin{array}{c} \text{MI}\left(A_{i},B_{j}\right)=H\left(A_{i}\right)+H\left(B_{j}\right)-H\left(A_{i},B_{j}\right), \end{array}$$

where *H*(*A*_*i*_) and *H*(*B*_*j*_) are the marginal probability distribution functions of the intensities *A*_*i*_ and *B*_*j*_ and *H*(*A*_*i*_, *B*_*j*_) their joint probability distribution function. This metric was used in conjunction with the original images and the original images filtered by the sigmoid filter and the two registration methods (demons and FFD).

For demons' method, the MSE-based implementation was the one from ITK [30], while the MI-based implementation was an in-house method [31].

For the FFD method, the implementation provided by the ElastiX library [32] was used.

Before the DIR step, all the weekly CT images were rigidly registered to the planning CT using a transformation defined by six parameters (the three translations and the three rotations). MSE was used with the descent gradient optimizer. This rigid registration was also included in the analysis, together with the different DIR methods.

Table 1 shows a total of 10 final DIR combinations or methods, each of them including one of the 2 image preprocessing methods (or no preprocessing), one of the 2 DIR methods, and one of the 2 metrics.

#### 2.4.4. Contour and Dose Distribution Propagation

The weekly dose distributions were propagated, using the 10 DIR and rigid registration methods, from the weekly CTs to the planning CT, with trilinear interpolation (Figure 1, step 3). For the evaluation, the contours obtained on the weekly CTs were also propagated.

### 2.5. Evaluation of DIR Geometric Performance

The DIR performance was evaluated using both volume-based criterion, that is, Dice similarity coefficient and locally by computing LM registration errors.

#### 2.5.1. Dice Similarity Coefficient (DSC)

The Sorensen-Dice is a similarity coefficient computed between two regions [33]. Its formula is

(6) $$\begin{array}{c} \text{DICE}=\frac{2*C}{A+B}=\frac{2*\left|A\cap B\right|}{\left|A\right|+\left|B\right|}, \end{array}$$

where *A* and *B* represent the two regions. *C* represents the number of shared voxels between the regions. Computed between the planning delineation of one organ and a weekly delineation propagated thanks to the deformation field, this coefficient expresses the overlap between the two regions: between zero (no overlap) and one (perfect overlap). The Dice similarity coefficient was computed for the following structures: the left and right PGs, the mandible, the spinal cord, the thyroid, the larynx, the brain stem, and the brain.

#### 2.5.2. Landmark Registration Error

Fourteen landmarks (LM) were defined, as described in Table 4: inside the left and right PGs, the posterior part of the intervertebral disk C2-C3, the superior and left part of the sternum (near the left sternoclavicula joint), the left and right carotid bifurcation, the left and right lesser cornu of the hyoid bone, the superior thyroid notch (part of the thyroid cartilage), the lower part of the mandible, the vallecula, the lower part of the palatine uvula, the philtrum, and the odontoid. LMs were defined by a first radiation oncologist expert on the planning CT. The same expert observer and a second expert observer defined afterwards the position of the LMs on the weekly CTs, in order to assess the interobserver variability.

The 14 LMs on CT0 were propagated to the six weekly CTs of each patient, for each of the 10 DIR methods and using the MSE rigid registration method for comparison. For each LM and for each weekly CT, the LM registration error of the different DIR methods was evaluated by computing the three-dimensional Euclidean distance between the positions of the LM as assessed by the first expert and as propagated by the deformation field estimated by DIR.

The DIR method performance was evaluated by the accuracy and precision parameters. The accuracy is defined by the average LM registration error, and the precision is defined by the average standard deviation of the LM registration error.

### 2.6. Dosimetric Impact of the DIR Methods for Dose Accumulation

To evaluate the dosimetric impact of the DIR errors, the reference cumulated dose was computed for each LM, by summing the weekly doses corresponding to the LM positions on the weekly CTs defined by the first expert. The same process was applied to cumulate the dose in the LM positions as assessed by the DIR methods, the rigid registration and by the second observer (interobserver variability). The cumulated dose errors were then calculated and defined by the differences between the LM reference cumulated dose (from the first expert) and each of the estimated LM cumulated doses (from the DIR, from the rigid registration methods and from the second expert). The planned dose corresponding to each LM has been also given for comparison (Figure 1, step 5). Such as for the LM registration error, the cumulated dose, estimated by each method and by the second observer, was evaluated by the accuracy and precision. The cumulated dose accuracy was calculated by the average of the difference between the reference cumulated dose (defined by the first expert for each LM) and the cumulated dose estimated by the different methods or the second observer. The cumulated dose precision was calculated by the standard deviation of the same difference.

The cumulated mean PG dose (considering the whole PG volume), clinically relevant since being highly correlated with the risk of xerostomia [34], was also computed for each DIR method, and finally also compared to the planned dose (Figure 1, step 5). Three PGs were excluded from the analysis since being included in the CTV_70_ and consequently not spared at the planning.

### 2.7. Statistical Analysis

The DIR methods evaluation was based on the DSC for the contour propagation and on the LM registration errors for the voxel-to-voxel precision. The interobserver variability was quantified by considering the LM distance between the first and the second expert observers. An analysis of variance (ANOVA) was carried out to assess the impact of each of the DIR methods (Table 1) on the DSC and LM error. Using ANOVA contrasts, all methods were tested against all methods. For all pairs (X, Y) of methods, the tested null hypothesis was H0: the mean error provided by “method X” is lower than the mean error provided by “method Y”, against the alternative hypothesis Ha: mean errors of “method X” and “Y” are equal. This procedure allows the identification of the DIR methods (or a subgroup of DIR methods) that leads to the lower mean error and also allows for intrapatient variability to be controlled.

Statistical analysis was carried out using the R language and environment for statistical computing.

## 3. Results 

### 3.1. Deformable Image Registration Geometric Performance

#### 3.1.1. Dice Similarity Coefficient for DIR Geometric Evaluation

**Figure 4:**
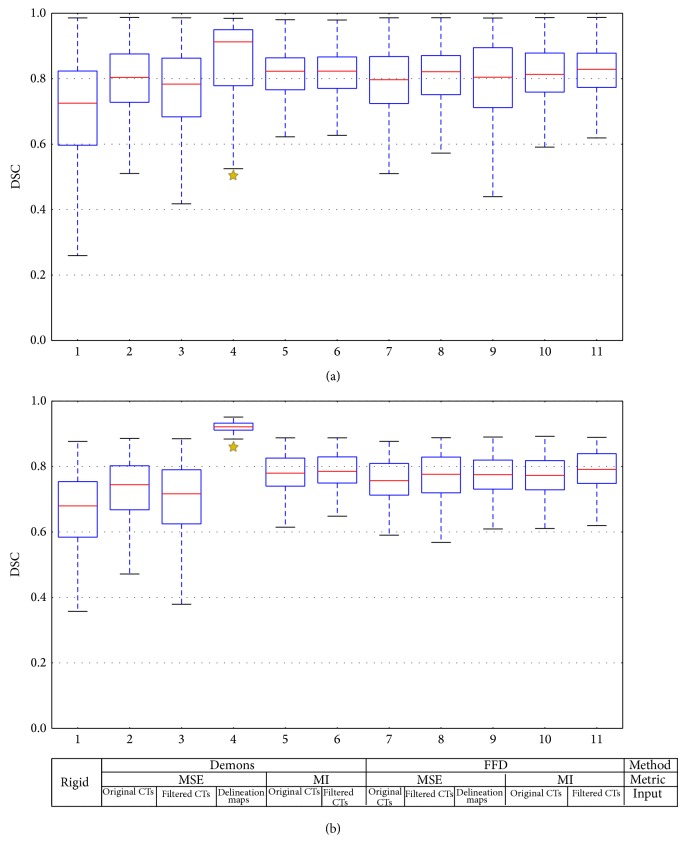
Boxplot of dice similarity coefficient by registration methods for (a) all the structures and (b) the parotid glands. DSC: dice similarity coefficient, FFD: free form deformation, MSE: mean squared error, MI: mutual information, and CT: computed tomography (scan). The limits of each box represent the 25th and 75th percentiles, the whisker represents the min and the max value, and the red line represents the median (50% of the total values). Each boxplot is represented without the outliers.

Figure 4 shows the DSC distribution corresponding to each of the 10 DIR methods and the rigid registration, for all the structures (Figure 4(a)) and the PGs only (Figure 4(b)).

- Considering all the anatomical structures, the three methods corresponding to the maximum DSC were the “demons with MSE on delineation maps,” the “FFD with MI on filtered CTs” and the “demons with MI on filtered CTs.” The corresponding median (standard deviation) DSC values were 0.91 (0.16), 0.83 (0.09), and 0.82 (0.09), respectively. The “FFD with MI on filtered CTs” DSC values were higher than the DSC values of all other methods (*P* ≤ 0.01), except the “demons with MSE on delineation maps” method. The “demons with MI on filtered CTs” DSC was superior to all DSC methods with MSE on original CTs (*P* ≤ 0.05), except the “demons with MSE on delineation maps” method.
- Considering only the PGs, the “demons with MSE on delineation maps” DSC values were higher than the DSC values of all other methods (*P* < 0.01). The “FFD with MI on filtered CTs” DSC values were superior to the DSC values of 7 among the 9 other DIR methods (*P* < 0.05).

Regardless of the anatomical structures, the DSC of all DIR methods was superior to the DSC of the rigid registration (*P* < 0.01).

#### 3.1.2. Landmark Registration Error

Table 2 shows the mean Euclidean distance between the positions of the landmark (LM) assessed by the first expert (reference) and the positions propagated by the 10 considered DIR methods, the rigid registration and the second expert, for each LM. The table orders the methods, from the minimum to the maximum distance. The minimum mean error (2.44 mm) corresponds to the “FFD with MI on filtered CTs” method, while the maximum error (5.16 mm) corresponds to the rigid registration.

The interobserver distance (between the positions of the LM assessed by the two expert observers) results to an average error representative of the accuracy (average standard deviation representative of the precision) of 2.01 mm (1.29 mm).

The two most effective DIR methods were the demons and the FFD, with both the mutual information (MI) metric and the filtered CTs as detailed below.

- Considering all the LMs, the corresponding accuracy (and precision) were 2.44 mm (and 1.30 mm) and 2.54 mm (and 1.33 mm), for each method, respectively. The minimal errors were 1.18 mm and 1.13 mm for the odontoid LM, respectively. The maximal errors were 5.35 mm and 4.58 mm for the right carotid bifurcation LM, respectively. Considering bone LMs only, accuracy (and precision) were 2.13 mm (and 0.99 mm) and 2.15 mm (and 1.16 mm), respectively. Considering soft tissue LMs only, they were 2.66 mm (and 1.60 mm) and 2.88 mm (and 1.49 mm), respectively. The “FFD with MI on filtered CTs” and the “demons with MI on filtered CTs” errors were inferior to 7 among the 9 other DIR method errors (*P* < 0.05).
- Considering only the PG LMs, the “FFD with MI on filtered CTs” and the “demons with MI on filtered CTs” errors were inferior to 7 among the 9 other DIR method errors (*P* < 0.03).

No method errors were statistically inferior to the interobserver variability error considering all LMs and the PG LMs.

#### 3.1.3. Impact of the Choice of the DIR Method on Dose Accumulation

Table 3 shows the cumulative dose error for each LM and for each method. The table shows also the planned dose at each LM and also the reference difference between planned and cumulated doses (from the first observer). The two last columns represent the accuracy and the precision of the methods defined by the average and the average standard deviation of the cumulated dose differences.

The median (SD) planned dose was 54.18 Gy (21.02 Gy) for all LMs, 43.20 Gy (6.17 Gy) for the C2-C3 LMs, and 48.18 Gy (18.20 Gy) for the PG LMs.

- Considering all the LMs, the methods corresponding to the lowest cumulated dose errors were the “FFD with MI on filtered CTs” and the “demons with MI on filtered CTs,” with corresponding accuracy (and precision) of: 0.85 Gy (and 0.93 Gy) and 0.88 Gy (and 0.95 Gy), respectively. The minimal errors were 0.36 Gy for the odontoid LM and 0.33 Gy for the sternum LM, for both methods. The maximal errors were 2.63 Gy and 2.74 Gy for the right PG LM, for each of the method, respectively. Considering bone LMs only, accuracy (and precision) were 0.50 Gy (and 0.49 Gy), and 0.49 Gy (and 0.48 Gy) for each of the method, respectively. Considering soft tissue LMs only, they were 1.20 Gy (and 1.38 Gy), and 1.27 Gy (and 1.41 Gy), respectively. The “FFD with MI on filtered CTs” errors were inferior to the errors of 6 among the 9 remaining DIR methods (*P* < 0.03).
- Considering the PG LMs only, the “FFD with MI on filtered CTs” and “demons with MI on filtered CTs” errors were inferior to the “demons with MSE on delineation maps” errors (*P* ≤ 0.03).

For all the LMs, all method errors except the “demons with MSE on delineation maps” were statistically inferior to the rigid registration errors (*P* < 0.01).

Considering all the LMs or only the PG LMs, no method performs better than the second observer.

**Figure 5:**
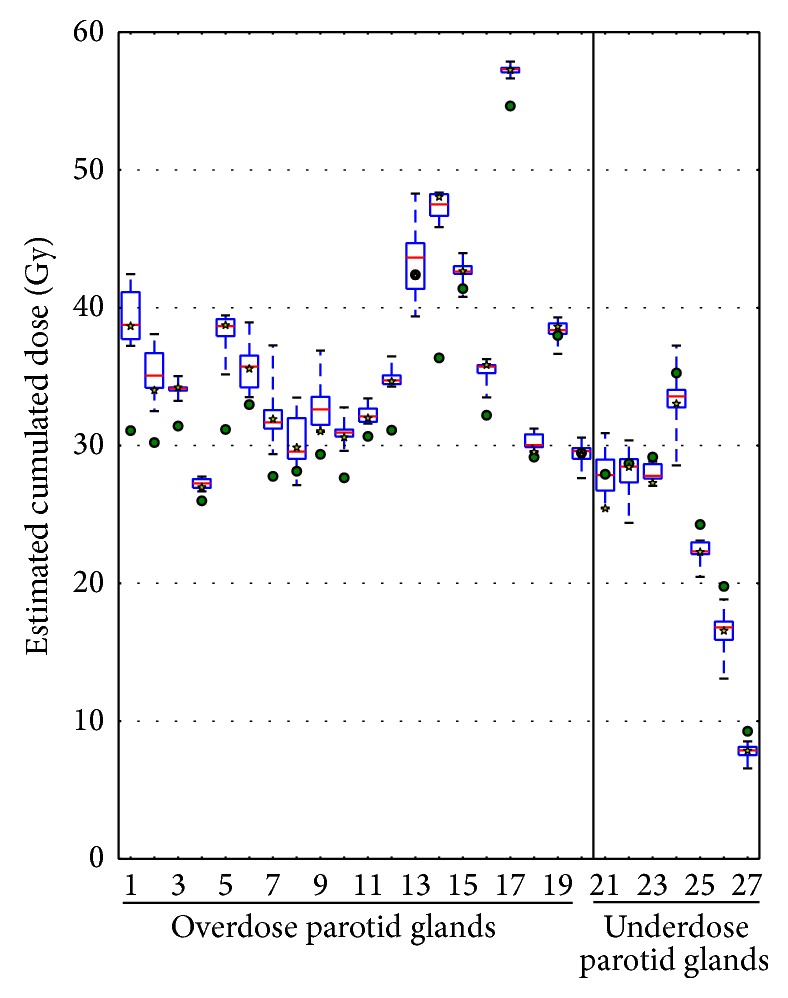
Variability between estimated cumulated dose by method for the same organ with the mean dose in the parotid gland (overdose and underdose). The green points represent the planned dose and the yellow stars represent the value returned by the free form deformation with mutual information metric on filtered CT-scans method. The limits of each box represent the 25th and 75th percentiles, the whisker represents the min and the max value, and the red line represents the median (50% of the total values). Each boxplot is represented without the outliers.

Figure 5 represents the estimated cumulated mean dose in the PGs by each method (with a star showing the result using the “FFD with MI on filtered CTs” method). The median (SD) mean planned dose for the PGs was 30.22 Gy (7.76 Gy). The median (SD) mean cumulated dose for the PGs was 32.62 Gy (9.19 Gy) by using the “FFD with MI on filtered CTs” method.

The mean uncertainty (difference between maximal and minimal estimated cumulated doses considering all the methods) to estimate the cumulated mean PG dose was 4.03 Gy (SD = 2.27 Gy, range: 1.06–8.91 Gy). Using the “FFD with MI on filtered CTs” method to calculate the cumulated mean PG dose, 66% of the PGs presented an increase of the mean dose of 3.38 Gy (SD = 2.82 Gy, range: 0.38–11.69 Gy), and 33% of the PGs presented a decrease of the mean of 1.52 Gy (SD = 1.08 Gy, range: 0.06–3.22 Gy), compared to the mean planning dose.

**Figure 6:**
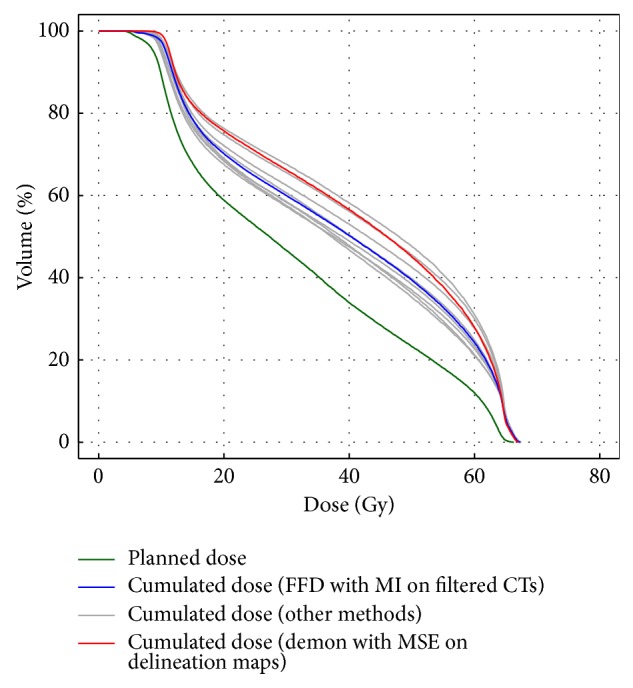
Example of DVHs variability in the estimated cumulated dose depending on the registration method (10 tested methods) for one patient (parotid gland number 1 of Figure 5). FFD: free form deformation, MSE: mean squared error, and CT: computed tomography (scan). The green DVH represents the planned dose. The blue DVH results from the free form deformation with mutual information metric on filtered CT-scans method. The DVH in red results from the demon with MSE on delineation maps method. The dark grey DVHs represent the other methods.

Figure 6 represents the DVHs of the planned dose and of the cumulated dose estimated by all the DIR methods for one patient (parotid gland number 1 of Figure 5). The blue line represents the DVH of the “FFD with MI on filtered CTs” method that has an overall average accuracy (precision) of 0.68 mm (0.75 mm) and an average DSC of 0.79 for the parotid glands. The red line represents the DVH of the “demon with MSE on delineation maps” method that has an overall average accuracy (precision) of 1.89 mm (2.14 mm) and an average DSC of 0.92 for the parotid glands.

## 4. Discussion

The goal of our study was to compare different registration methods with regard to their ability to monitor the accumulated dose in HNC radiotherapy, mainly in deformable anatomical structures and using CT-scans. Evaluating 10 DIR methods with 14 landmarks, we found that the two most accurate methods were the demons and the FFD, both combined with the mutual information metric and filtered CTs. Their estimated registration accuracy and precision were 2.44 mm and 1.30 mm, respectively, with an improvement on bony structure against soft tissues up to 0.87 mm. The corresponding LM estimated cumulated dose accuracy and precision ranged from 0.50 to 0.95 Gy. Those corresponding errors were significantly inferior to the rigid registration errors by a difference of accuracy and precision for all the LMs of 2.72 mm and 1.22 mm, respectively, with a similar impact of 1.34 Gy and 1.67 Gy on the estimated cumulated dose.

Our observed volume diminutions from the beginning to the end of treatment were 28% for the PGs and 31% for the CTV_70_, in concordance with the literature reporting values of 15% to 28% for the PGs, 69% for the GTV, and 8% to 51% for the CTV [1, 3, 11, 12, 19, 35]. The dosimetric impact of such anatomical variations is particularly important for organs located close or within high dose gradients, such as the PGs, whose overirradiation leads to xerostomia decreasing strongly the patient quality of life [6, 7]. In this context, the ability of dose monitoring by cumulating the dose, fraction after fraction, is an important step for the implementation of the dose-guided ART strategy. Indeed, the replanning decision should be optimally taken by considering the dose actually delivered to the tissues from the beginning of the treatment, and by comparing this cumulated dose with the planned dose. The quantification of the accuracy of the DIR methods to cumulate the dose in deformable structures appears therefore particularly crucial to choose the most appropriate one and make consequently the whole ART approach clinically feasible.

Weekly CT-scans were used in our study since CBCTs cannot be considered for an accurate dose calculation, both at the fraction and for replanning. Moreover, performed just before or after the fraction, the CTs provide a representative anatomy of the irradiated patients with a small intrafraction anatomical variation. All the planning CTs were however acquired with a contrast agent injection, while the weekly CTs were mostly acquired without injection due to the renal toxicity risk of the cisplatinum, concomitantly given with the radiotherapy. This makes the registration particularly challenging since some tissues such as the vessels and the PGs present therefore different intensity values in the registered images.

We evaluated the two most popular registration approaches, demons, and FFD methods, combined with two metrics and two kinds of preprocessing methods. We showed that, more than the used DIR approach (demons or FFD), the considered preprocessing and metric have a significant impact on the results. As expected, the incorporation of the delineation maps with demons' method enables to increase the DSC, especially for the organs used in the delineation maps (e.g., PGs). Using the delineation maps with the FFD approach is not really efficient, since it uses a grid of control points, which may not lie on the organs boundaries, that is, the salient information. Our results can be compared with the study of Castadot et al., analyzing 12 voxel-based DIR strategies with a dataset of 5 HNC patients imaged with CT before and once during RT [15]. Considering the DSC, the best three strategies were the demons' algorithm implemented in multiresolution, the multiresolution demons combined with level-set, and a denoising filter and the multiresolution demons combined a denoising filter. Our DSC values (0.83) are comparable or slightly inferior using our best method without using delineations (“FFD with MI on filtered CTs” method), compared to the 0.86 value obtained by the “demons MSE” method in the Castadot study [15]. Our study comprised however more patients and more images per patient, and all the images were contrast-enhanced in Castadot study. The DSC, as other region-based criteria (e.g., Jaccard index or Hausdorff distance), is useful to globally evaluate the overlap between regions and thus, to evaluate DIR when it is used for delineation propagation. However, for a local dose accumulation purpose, an evaluation of the local point to point matching is needed. Indeed, the method performance is different when considering a local matching evaluation provided by the LM registration errors. Our two best DIR methods are the FFD and the demons, with both MI and sigmoid filter. Thus, even if the considered images are monomodal, the intensity differences caused by the contrast agent injection are better taken into account by the MI metric. The incorporation of the sigma-filter enables also to improve the results by enhancing the soft tissues contrast relatively to the other tissues which naturally present a very high contrast (especially bones and air). The mean distance LM registration error was 2.44 mm for the “FFD with MI on filtered CTs” method. It has to be compared to the second observer mean error which was 2.01 mm, showing a high interobserver variability. Moreover, considering the spatial resolution of the considered images (mean resolution of planning CTs: 1.10 × 1.10 × 2.4 mm^3^), this mean error corresponds to 0.70 times the slice thickness. It should also be noticed that all the LMs do not present equivalent errors, showing that some organs are easier to register than some others. These facilities could be related to the volume of the organs, where it could be hard to overlap small organ and preserve the point to point accuracy. The LMs located on bony structures globally present lower registration errors than those located on soft tissues, especially on the carotids' bifurcations (3.85 mm for the “FFD with MI on filtered CTs” method).

When considering the dose accumulation errors, the methods ranking remains the same, with the best results provided by considering MI and sigmoid filtering. It is important to notice that the geometrical uncertainties may have very different impact on the cumulated dose errors, depending on the value of the dose gradient on the considered area: a high geometrical error located in an area with a homogeneous dose distribution has a small impact, while even a small geometrical error may lead to high cumulated errors if it is close to a high dose gradient. For example, the “FFD with MI on filtered CTs” method, providing a mean geometrical error of 3.85 mm and 2.64 mm for the carotid bifurcations and PGs, respectively, results in a mean cumulated dose error of 0.69 Gy and 2.52 Gy, respectively. It shows that, when considering local dose accumulation, the results should be considered very carefully, especially for regions with a low contrast and localized close to high dose gradients such as the PG. However, the uncertainties resulting from DIR are lower than the difference between the planned and the delivered doses at the position of the LMs, showing that DIR could be useful to detect over- or underirradiation. This is notably true when considering dose descriptors, such as the mean dose delivered to the PGs. The DIR methods enable to detect potential deviations from the planned dose. As shown in Figures 5 and 6, even considering the uncertainties carried by the different registration methods, it is possible to confidently identify the globally under- and overirradiated PGs, the mean uncertainty (difference between maximal and minimal estimated cumulated dose considering all the methods) being 4.03 Gy.

Using the “FFD with MI on filtered CTs” method to cumulate the mean dose in the PGs and in comparison with the planning dose, 67% of the PGs were found to be overirradiated to a mean dose of 3.4 Gy (up to +11.7 Gy for one patient), justifying particularly the use of ART in the majority of locally advanced HNC. On the other hand, surprisingly, 33% of the PGs presented a decrease of the mean PG dose of 1.52 Gy (up to −3.2 Gy for one patient).

Figure 3 shows the impact of the anatomical variations on the dose distribution at the fraction (normalized to 70 Gy). The DVH differences for the CTV are very limited since the CTV is well treated both at the initial planning and during the treatment due to the tumor shrinking and to the CTV-to-PTV margins. On the other hand, for the parotid glands, the DVH differences along the DVH are ±10% for 75% of the fractions. For the spinal cord and the brain stem, the DVH differences are inferior to the parotid gland and below 45 Gy except for one patient who had large anatomical and position variations.

Such variations in the difference between the planned and the delivered PG doses justify the development of tools to target the right patients benefiting the most of the ART strategy. These tools can be based on a cumulative dose estimation or more simply, likely on the identification of anatomical markers ideally on the CBCTs, and early enough in the course of treatment to propose one or several replanning. Such markers could be the neck diameter, whose diminution increases the risk of overirradiation [4, 5].

One limitation of this work is to consider the manual expert observer as the reference. As shown by the second observer results, the interobserver variability is high, especially on low contrast regions. Previous studies proposed the use of finite-element numerical phantoms [31, 36] in order to generate automatically organ deformation with known point to point anatomical correspondence. However, generating phantoms with realistic deformations is particularly challenging, especially for the HNC localization, which contains small structures and presents complex anatomical deformations such as melting of the tumor and PG deformation. Another method for DIR evaluation is to replace the expert observer by an automated process to identify salient points in the registered images to compute landmark registration errors, as published by Paganelli et al. [37], with scale invariant feature transform (SIFT) descriptors. Moreover, if the two main registration methods have been evaluated (demons and FFD), other methods should be evaluated, such as the level-set method [15] or salient-feature-based registration [38]. Another limitation is related to the considered images. The obtained results would be different if considering only contrast-enhanced or only non-contrast-enhanced images, or CBCT images. Finally, in a dose accumulation purpose, we only evaluated the impact of DIR, and not in particular the dose mapping method. In this study, we used the “classical” trilinear interpolation but other methods, such as energy/mass mapping [39] have been proposed.

Finally, we did not evaluate in this work the capability of DIR methods to adequately describe tumor response during treatment. The account of disappearing tumor is indeed particularly challenging by using DIR methods. Nithiananthan et al., in a context of CBCT-guided surgical procedures, shown however, that adaptation of the demons deformable registration process to include segmentation (i.e., identification of excised tissue) and an extra dimension in the deformation field provided a means to accurately accommodate missing tissue between image acquisitions [40].

To further investigate this issue of the DIR performance in the presence of tumor variation, Mencarelli et al. used implanted small gold markers around the tumor, representative of a part of the tumor border, and in 13 oropharynx carcinomas [16]. Like in our study, they avoided the ground truth problem by computing the DIR algorithm performance with a measure relative to two observers. Otherwise, even by reducing the interobserver variability, the performance of their method was comparable to our best method with an accuracy and a precision of 2.2 mm and of 0.59 mm, respectively. However, they did not measure the impact of their DIR method performance in term of dose accumulation, especially in the high gradient zone. The conclusion of this recent study was that the B-spline-based DIR method was not capable of capturing the displacement of tumor borders, and that precision degraded during the course of treatment. Consequently, the use of DIR for specifically tumor dose accumulation in HNC cannot be applied for ART.

## 5. Conclusion

In this study, we evaluated 10 DIR strategies for dose accumulation in the context of IMRT for locally advanced HNC. We showed that the choice of the metric or of the image preprocessing was at least as important as the registration method. The best results were obtained by demons' and FFD methods, with both MI and filtered CTs. We showed that, if the estimated local accumulated dose has to be considered carefully, the most accurate methods provide the means to detect over- or underirradiation for healthy tissues.

## Figures and Tables

**Table 1:** Summary of all the 10 tested DIR methods, comprising a combination of one registration method, one preprocessing or not CT images and one metric.

		Rigid MSE	Demon MSE	Demon MSE Filtered CTs	Demon D. Maps	Demon MI	Demon MI filtered CTs	FFD MSE	FFD MSE filtered CTs	FFD D. maps	FFD MI	FFD MI filtered CTs
Registration method	Rigid	X										
	Demons		X	X	X	X	X					
	FFD (B-Spline)							X	X	X	X	X

Input data	Original CTs	X	X			X		X			X	
	Filtered CTs			X			X		X			X
	Delineation Maps				X					X		

Metric	Mean squared error (MSE)	X	X	X	X			X	X	X		
	Mutual information (MI)					X	X				X	X

A rigid method has been also evaluated for the comparison.

FFD: free form deformation, MSE: mean squared error, MI: mutual information, and CT: computed tomography (scan).

**Table 2:** Average 3D Euclidean distance (mm) by landmarks and by registration methods with the first observer as anatomical reference.

	Landmark Distance Error (mm)												
	Bones						Soft tissues					Precision (AVG)	Accuracy (AVG SD)
	1	2	3	4, 5	6	7	8	9	10	11, 12	13, 14		
**Interobserver variability**	1.00	1.24	1.40	2.21	2.43	2.56	1.27	1.36	1.85	2.19	3.15	2.01	1.29
FFD MI filtered CTs	1.18	2.60	1.45	1.62	3.03	2.89	2.64	2.07	2.11	3.85	2.64	2.44	1.30
Demons MI filtered CTs	1.13	2.40	1.72	1.77	2.93	2.97	2.27	2.84	2.89	3.63	2.79	2.54	1.33
Demons MI	1.13	2.36	1.80	1.82	3.03	3.12	2.36	2.79	2.87	3.79	2.82	2.59	1.38
Demons MSE	1.15	2.79	1.81	1.65	3.42	3.21	3.02	2.72	2.58	3.82	3.84	2.81	1.63
FFD MSE filtered CTs	1.32	3.49	1.62	1.91	3.50	3.27	3.63	2.94	2.19	4.06	3.10	2.86	1.54
FFD MI	1.07	3.81	1.93	1.77	3.37	3.52	3.96	3.11	2.07	4.16	3.07	2.92	1.60
Demons MSE filtered CTs	1.35	3.05	1.80	1.82	3.60	3.63	3.13	2.78	2.92	3.80	4.25	3.00	1.64
FFD MSE	1.83	5.13	3.30	2.50	4.07	4.37	5.14	5.05	2.63	5.42	3.48	3.88	2.18
Demons D. maps	2.43	5.51	4.62	2.79	4.40	4.75	5.17	5.97	4.01	6.35	3.41	4.43	2.14
FFD D. maps	2.40	6.03	4.02	3.15	4.42	4.80	5.94	6.37	4.37	6.68	3.55	4.65	2.33
Rigid MSE	3.16	6.26	4.32	4.16	4.85	5.11	6.26	6.57	4.69	6.85	4.53	5.16	2.52

AVG: average, AVG SD: average standard deviation, FFD: free form deformation, MSE: mean squared error, MI: mutual information, and CT: computed tomography (scan).

Methods are classified by their performance order. The performance is defined by the accuracy (average Euclidean distance error) and the precision (average of the standard deviation Euclidean distance error). A second observer allows quantifying the interobserver variability.

The “FFD with MI on filtered CTs” errors are inferior to all the methods errors (*P* < 0.05), except for the “demons with MI on filtered CTs” and “demons with MI on original CTs” methods (*P* > 0.15). The “demons with MI on filtered CTs” errors are inferior to all the methods errors (*P* ≤ 0.03) except for the “demons with MI on original CTs,” and the “FFD on filtered CTs” for both MSE and MI metrics (resp., *P* = 0.24, = 0.08 and = 0.76).

**Table 3:** Average cumulated dose (Gy) error by landmarks and by registration methods with the first observer as anatomical reference.

	Landmark dose (Gy)															
	Bones							Soft tissues							Precision (AVG)	Accuracy (AVG SD)
	1	2	3	4	5	6	7	8	9	10	11	12	13	14		
**Planned dose**	44.47	35.13	54.87	64.91	63.64	8.84	44.50	64.74	21.60	66.87	66.91	63.95	39.71	41.37	48.68	10.35
**Cumulated dose difference** ^*^	1.95	1.96	1.68	1.96	1.71	3.09	2.87	2.55	2.52	1.93	0.69	1.12	5.17	4.25	2.39	2.40

** **	Landmark Cumulated Dose Error (Gy)															
**Interobserver variability**	0.41	0.23	0.29	0.38	0.67	0.26	0.28	0.31	0.41	0.77	0.14	0.61	1.68	3.14	0.68	0.75
FFD MI filtered CTs	0.36	0.60	0.43	0.58	0.69	0.43	0.41	0.59	0.63	0.74	0.91	0.48	2.63	2.41	0.85	0.93
Demons MI filtered CTs	0.40	0.39	0.60	0.57	0.71	0.33	0.45	0.41	1.09	0.75	0.69	0.78	2.74	2.46	0.88	0.95
Demons MI	0.33	0.39	0.57	0.54	0.69	0.38	0.48	0.47	1.11	0.90	0.73	0.80	2.56	2.33	0.88	0.92
Demons MSE	0.41	0.52	0.42	0.48	0.88	0.40	0.43	0.56	0.85	1.38	0.74	0.75	3.15	2.96	0.99	1.22
FFD MSE filtered CTs	0.49	0.69	0.52	0.59	0.86	0.55	0.38	0.83	0.95	0.83	0.98	0.57	2.37	3.09	0.98	1.09
FFD MI	0.31	0.63	0.47	0.53	0.53	0.48	0.61	0.82	1.09	0.84	0.85	0.85	2.45	2.68	0.94	1.02
Demons MSE filtered CTs	0.41	0.52	0.42	0.48	0.88	0.40	0.43	0.56	0.85	1.38	0.74	0.75	3.15	2.96	0.99	1.25
FFD MSE	0.34	1.16	1.13	1.27	1.02	0.45	0.92	1.34	1.88	1.26	1.06	1.86	2.75	3.91	1.45	1.60
Demons D. maps	0.99	1.29	3.11	1.19	1.24	0.53	0.94	1.28	1.76	2.39	1.29	2.22	3.05	5.14	1.89	2.14
FFD D. maps	0.95	1.56	1.24	0.94	1.53	0.53	1.00	1.54	2.66	2.90	1.32	2.54	2.99	4.87	1.90	2.18
Rigid MSE	0.94	1.67	1.20	2.78	1.80	0.59	1.02	1.71	3.35	3.99	1.35	2.61	3.44	4.27	2.19	2.60

AVG: average, AVG SD: average standard deviation, FFD: free form deformation, MSE: mean squared error, MI: mutual information, and CT: computed tomography (scan).

Methods are classified by their performance order. The performance is defined by the accuracy (average of the cumulated dose error) and the precision (average of the standard deviation cumulated dose error). The cumulated dose difference (∗) represents the reference difference between planned and cumulated doses from the first observer. A second observer allows quantifying the interobserver variability.

The “FFD with MI on filtered CTs” errors are inferior to all the methods errors (*P* < 0.03), except for the “demons with MI on filtered CTs” and “demons with MI on original CTs” methods and for the “demons with MSE on original CTs” (*P* = 0.06). The “demons with MI on filtered CTs” errors are inferior to the “delineation maps based method” errors, the “demons with MSE on filtered CTs” errors, and the “FFD MSE on original CTs” errors (respectively, *P* < 0.01, *P* ≤ 0.03 and *P* < 0.01).

**Table 4:** List of all the 14 landmarks used for the study.

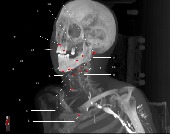		

Landmark index	Tissue landmark	Description

1	Bone	The odontoid
2		The lower part of the mandible
3		The superior thyroid notch (part of the thyroid cartilage)
4-5		The right (4) and left (5) lesser cornu of the hyoid bone
6		The superior and left part of the sternum (near the left sternoclavicular joint)
7		The posterior part of the intervertebral disk (C2-C3)

8	Soft	The vallecula
9		The philtrum
10		The lower part of the palatine uvula
11-12		The right (11) and left (12) carotid bifurcation
13-14		The right (13) and left (14) parotid gland (PG)

A total of 7 bony landmarks and 7 soft tissue landmarks have been defined.
